# Advancing real-time validation of automotive software systems via continuous integration and intelligent failure analysis

**DOI:** 10.1038/s41598-025-21416-5

**Published:** 2025-09-25

**Authors:** Mohammad Abboush, Christoph Knieke, Andreas Rausch

**Affiliations:** Institute for Software and Systems Engineering, Technical University of Clausthal, 38678 Clausthal-Zellerfeld, Germany

**Keywords:** Engineering, Electrical and electronic engineering

## Abstract

In the automotive industry, a rigorous testing process based on ISO 26,262 is carried out at various stages of the V-model to ensure the quality of software systems. Conventional validation of embedded electronic control units (ECUs) using hardware-in- the-loop (HIL) testing is performed in the late stages using the big bang integration style, resulting in delayed feedback, lack of scalability, and insufficient fault diagnosis. Furthermore, test recording analysis is performed manually based on expert knowledge to identify the nature of the failure occurring. This, in turn, resulted in higher development costs and effort, delays fault detection, and hinders agile collaboration. To address these gaps, this article proposes a novel continuous integration (CI)-enabled HIL testing framework to facilitate continuous software development through iterative cycles. Furthermore, based on a representative critical faults dataset, intelligent data-driven ML-assisted Fault Detection and Diagnosis (FDD) models are developed, including LSTM and K-means for the diagnosis of known and unknown sensor-related faults as classification and clustering problems, respectively. The novel aspect of the robust models lies in the integration of a denoising autoencoder (DAE) for the extraction of representative features before the classification and clustering process, considering the noise conditions. The evaluation outcomes illustrate the superiority of the proposed model for known faults classification in comparison to other state-of-the-art methods, with an average F1-score of 91.85%. Furthermore, the integration of DAE with k-means exhibited a high clustering performance against noise with a low mean squared error (MSE) and Davies–Bouldin index (DBI), i.e., 0.044 and 0.68, respectively. It has been demonstrated that the proposed methodology facilitates more efficient, automated, and accurate fault analysis within the framework of automotive software validation workflows. Consequently, this approach enhances both safety and efficiency in comparison to conventional methodologies.

**Supplementary Information:**

The online version contains supplementary material available at 10.1038/s41598-025-21416-5.

## Introduction

 This article is an extension of our previous publication, which proposed an intelligent approach for failure analysis in test records resulting from the real-time validation process of automotive software systems (ASSs)^1^.

In accordance with the ISO 26,262 functional safety standard, the requirements for the development process of automotive systems have been defined. These requirements encompass the systematic, reproducible and timely validation of safety-related software systems^2^. However, the growing complexity of the functions within these systems has resulted in the emergence of additional technical challenges with regard to design, implementation, testing and maintenance^3,4^. To overcome these challenges during the development cycle with frequent code changes and short release cycles, continuous integration (CI) with automation and rapid feedback has become a standard practice in the software development process^5^. Operating as an agile software development method, CI enables developers to frequently integrate, build, and test software, thereby ensuring the required level of safety and reliability while delivering products on time^6^. Although the CI approach has gained significant traction within the automotive industry, its implementation is predominantly limited to purely software-controlled systems in the initial phase of the V-model, specifically for software-in-the-loop verification^7^.

In the system integration phase of the V-model, hardware-in-the-loop (HIL) testing is strongly recommended to validate the behaviour of the system under test on the target physical machine in real-time^8^. However, extending traditional CI-based validation frameworks to the HIL environment poses a significant challenge in terms of scalability, adaptability, and fault analysis process. In particular, the involvement of hardware components and the limitation of access to shared resources in HIL testing frequently result in bottlenecks during test execution, thereby negatively impacting the feedback cycle for developers. Moreover, the present CI framework, which is optimised for asynchronous and non-real-time builds, is incapable of meeting the requirements for deterministic timing during test execution. Furthermore, standard CI pipelines are incapable of capturing, storing, and analysing the substantial quantity of logs, sensor traces and records of HIL testing. Conventionally, test records are analysed either by certified software tools with solely binary pass/fail results or by knowledge-based analysis approaches. However, on the one hand, the industrial tools are limited in their ability to identify the anomaly in the observations, and thus lack the capacity to identify the nature and causes of the occurring failures. It is important to note that the anomaly may be caused by external factors and not necessarily a critical fault that leads to unintended risks^9^. Furthermore, uncertainty in the dataset, including noise, can also be a complicating factor during the analysis process^10^. On the other hand, besides the necessity of expert knowledge, the manual inspection of test records is associated with a high expenditure of time, labor and costs^11^. Moreover, as the interpretations of the evaluation are contingent upon human experience, the process is susceptible to error in certain instances, such as the occurrence of simultaneous faults, which may result in inconsistencies in fault detection. Consequently, the development of advanced technology-assisted failure analysis is of paramount importance for the identification of potential risks and their causes during the validation process in an efficient manner. In order to achieve this objective, a variety of methodologies have been utilized in the literature for the development of fault detection and diagnosis (FDD) models. The knowledge-based approach^12^, signal processing-based approach^13^, and data-driven approach^14,15^ are three widely used approaches in various engineering domains.

In the past decade, the availability of sufficient computational resources has facilitated the widespread application of a data-driven approach to the problem of FDD. This approach is based on the extraction of knowledge from historical data and the construction of non-linear relationships between input and output classes, with the aim of discovering hidden patterns. In comparison to other approaches, it does not require the input of expert knowledge or the use of a precise mathematical model. Deep learning (DL) and machine learning (ML)-based methods, which are a subset of the data-driven approach, are widely used in different phases of the software development life cycle^16^. Notable accomplishments have been achieved by ML/DL-based models for various engineering challenges, including those related to fault detection, clustering, classification, and regression^17^. Among the mentioned structures, autoencoder (AE), Generative adversarial network (GAN), Convolutional neural network (CNN), Deep belief network (DBN), and Recurrent neural network (RNN) have been widely investigated^18^. In the same regard, graph neural networks (GNN)-based methods have demonstrated a pivotal role in fault detection, a feat attributable to their resilience against noise and model dependencies^19^. The fundamental premise of this approach is to utilise graph representations in a manner that reflects the relationships between system components and the dependencies between data instances. Among these methods, GCI-ODG and EGN-OOD have demonstrated the capacity for effective fault detection, taking into account subtle interdependencies between system components when data patterns may be subject to change due to noise, environmental factors, or unexpected fault combinations^20^. However, the current state-of-the-art FDD models based on a data-driven approach are inadequate in several respects. This includes their inability to identify concurrent faults under imbalanced datasets^21^. Moreover, the challenge of detecting and categorizing unknown faults in the presence of noise requires further investigation^22^. It is notable that the absence of representative real-time datasets of critical faults represents a significant obstacle to the development of FDD models in both aforementioned tasks^23^.

In light of the aforementioned limitations, this article aims to address the identified gap by developing CI pipelines to incorporate a real-time validation process for safety-critical systems. The proposed research constitutes a novel CI-based validation framework that integrates HIL simulation and an intelligent failure analysis process, thereby enabling a scalable and adaptive validation process involving real hardware components. Furthermore, to address the challenges of test recordings analysis considering the noisy conditions, two intelligent models based on Long Short-Term Memory (LSTM) and k-means techniques were developed. As a preliminary stage, Denoising Autoencoder (DAE) was developed to ensure comprehensive representative feature extraction for efficient classification and clustering processes. The proposed methodology contributes to the field of real-time validation of ASSs, thereby enhancing the safety and reliability of the system while reducing the cost and effort required during the development process. To the best of our knowledge, this is the first study to investigate the applicability of integrating an intelligent FDD and CI practices during the system integration and testing phase of the V-model.

The main contributions of the proposed study can be summarized as follows:

- To enable a rapid response to changes and to facilitate continuous software development, the CI approach was integrated into the HIL test activities, allowing efficient change tracking and traceability during the real-time validation process.
- To address the challenge of detecting and classifying the known simultaneous faults in the test automation phase of CI pipeline, LSTM-based model is developed and evaluated.
- To detect and cluster the unknown single and concurrent faults, K-means clustering has been adopted.
- To ensure a high-quality classification and clustering process with robust resistance to noise, GRU-based DAE was developed for extracting detailed representative features.
- To provide a representative faults dataset, a real-time fault injection (FI) framework is utilized. This involves identifying the critical faults and collecting a related dataset through a process of analyzing the system’s behaviour under faults.
- To assess the performance of the developed models, a real-time dataset derived from a high-fidelity simulation-based virtual test drive platform was utilized.

The remainder of the paper is structured as follows. The related works are reviewed and discussed in Section II, highlighting the gaps and differences between our study and other works. The proposed approach is presented in Section III, including the main phases and process. The implementation setup and case study are described in Section IV. The results and findings are discussed and summarized in Section V. Finally, the conclusion and future work are presented in Section VI.

## Related work

In this section, the relevant studies are reviewed and discussed, thereby demonstrating the differences between the proposed approach and other studies and highlighting the gap in the literature.

### Machine learning-based intelligent failure analysis

In the past decade, data-driven DL methods have demonstrated their capacity to provide reliable and efficient FDD models without the necessity for prior expert knowledge. Moreover, the advent of sophisticated sensor technology and the availability of substantial computational resources have facilitated the development of sophisticated FDD systems by researchers for a range of engineering applications, including classification, regression, and clustering^17^. Nevertheless, the unavailability of representative historical time series data remains a significant challenge in certain domains, including automotive, railways, and aviation. In the automotive domain, to overcome this challenge, researchers primarily rely on various sources of datasets, including online public industrial datasets, real vehicle prototype datasets, and simulation datasets. For instance, Safavi et al. in^24^ have proposed a DL algorithm to perform detection, isolation, identification, and fault prediction of ASSs based on the time sensor signals dataset offered by Audi AG. The proposed approach demonstrates high performance in terms of accuracy, with a detection accuracy of 99.84% and identification accuracy ranging from 73.00% to 100.00%. However, due to the fact that the data has been collected during a real test drive, the majority of the sensor-related fault types have not been covered. In contrast, our approach employs a real-time FI method to generate a representative faults dataset covering the majority of sensor/actuator-related faults, which serves as the basis for developing the target FDD model. In the same context, an ensemble classifier has been developed in^25^ based on ML techniques and the majority voter to analyse the recordings of the real test drives. Specifically, MOG, naive Bayes, SVM, and random forest have been employed to develop a two-class classifier, whereas extreme value, Mahalanobis, OC-SVM, and SVDD have been used to develop a one-class classifier. Nevertheless, despite the remarkable success of the proposed algorithm, the analysis process of the recordings was limited to anomaly detection. In contrast, in our study, sensor-related fault type identification was also covered. Similarly, an intelligent FDD model of an electromechanical conversion chain in an EV based on LSTM has been proposed by^26^. The validation results of the proposed approach, based on real vehicle prototype testing data, demonstrate its capability for FDD of multiple types of electrical faults. However, it should be noted that the FI method has been employed in the Simulink model of the EV to generate the training data without consideration of the real-time constraints. Consequently, the developed FDD model lacks reliability for real-time applications during the operation phase. Similarly, Biddle et al. in^27^ have developed an accurate SVM-based model for detecting, identifying, and isolating sensor-related faults of autonomous vehicles using faulty data generated from the MATLAB/IPG CarMaker co-simulation platform.

The recent advancement of the HIL simulation system in conjunction with FI proved to be a pivotal element in the provision of a high-quality, representative dataset of faults for AI applications in real-time^28^. The authors in^29^ have investigated the applicability of the Random Forest (RF) technique for developing a single fault identification scheme for the air brake system as a multiclass classification problem, depending on the aforementioned platform. The comparative results of six multiclass ML models demonstrated the superiority of the RF in terms of performance, with an accuracy of 91.99%. In contrast to the aforementioned study, this work encompasses not only single faults but also concurrent faults that occur simultaneously in different locations.

Due to the critical impact of the simultaneous occurrence of faults at the system level^30^, researchers have shed light on the importance of addressing this issue. For instance, Li et al.^31^ have proposed a novel FDD approach based on the multi-label classification strategy that is able to identify the concurrent faults of solid oxide fuel cell (SOFC) systems using only a dataset of single faults. Specifically, the developed model covers nine different single faults of air and fuel leakage, as well as three combinations. The validation results of the proposed multi-class SVM model demonstrate the effectiveness of single FDD, with an F1-score of 96.4%. Nevertheless, the proposed model’s performance for simultaneous faults, as indicated by the F1-score of 84.93%, necessitates further enhancement. Wong et al.^32^ have proposed a probabilistic committee machine-based FDD model for the diagnosis of simultaneous automotive engine faults. The proposed diagnostic framework is distinguished by its ability to diagnose 15 types of faults with an accuracy of 92% and 81.49%, respectively, when occurring individually or simultaneously. In contrast to the aforementioned study, our study demonstrates the ability of the model to perform simultaneous fault detection in the presence of noisy data, as well as to identify single and compound faults in time-series data.

In summary, while there has been significant progress in the field of developing data-driven DL-based FDD models for engineering problems, further investigation is needed in the analysis of test records to detect and identify single and.

simultaneous faults under noisy conditions. The proposed study represents a novel approach for addressing the aforementioned limitations based on LSTM and DAE-k-means. It entails the development of an intelligent model with high robustness against noise based on a historical, real-time, representative critical faults dataset generated by FI during the validation process. The proposed ensemble learning approach is capable of detecting and identifying both known and unknown faults, which may occur individually or simultaneously in ASSs.

### Continuous integration practices and HIL testing

The application of CI in the domain of automotive systems development has recently garnered significant interest from both academic and industrial research communities. The rationale behind this can be attributed to the noteworthy accomplishments of the iterative workflow of CI practice in mitigating development risks and enhancing product quality. At the vehicle level, for example, the first attempts to investigate the CI strategies in the automotive industry were made by Vöst in^33^. In this study, an adapted process of holistic CI was presented, focusing on the specification and selection of test cases. While the study incorporates the HIL concept, enabling software tests to be executed on real vehicle electronic components, it lacks a pronounced emphasis on CI server architecture and pipeline design. In contrast to the study, which focuses on the development of effective test suites and the dynamic selection of relevant tests, our work makes a greater contribution to automating the test evaluation process based on DL methods, thereby reducing the manual testing effort. In a similar context, Reiterer et al.^34^ investigated the applicability of the CI approach to simulation components in a large co-simulation environment. The contribution of this work lies in the ability of the proposed framework and tool to automatically generate the required CI pipelines based on a graph-based metadata model, thereby reducing the effort required to write software code. However, the real-time validation process of the system under test (SUT) on the target embedded machine was not considered in the proposed work. Our proposed CI-based validation framework, on the other hand, targets the system integration level of the V-model, where the physical hardware component of the SUT is connected to a HIL real-time simulator. Kaijser et al.^35^ propose a workflow and system representation approach for applying continuous integration and continuous deployment (CI/CD) activities with simulation-based verification. The proposed approach encompasses three distinct aspects, i.e., system representation and variability, simulation fidelity, and verification and validation (V&V) aspects. However, the proposed study does not address the interaction of the SUT with hardware components. Whilst the study’s objective is to automate the virtual verification of the integrated system in general, the HIL system is considered the core of our proposed framework for meeting the requirements of ISO 26,262. Similarly, but focusing on a different aspect, Fu et al.^36^ investigated the occurrence of failures during the automated CI processes with general HIL testing. A study yielded results indicating that the software-related issues, such as configuration problems, pipeline scripting, and dependency errors, are a primary cause of CI system failures. Conversely, the failure analysis process in our proposed framework encompasses unpredictable random hardware failures that occur in target systems, i.e., sensor/actuator-related failures. Finally, the challenges of incorporating CI for automotive software development in multi-vendor environments and their causes have been investigated in^37^. The work focuses on investigating the challenges, causes and possible solutions for the organisational and process-oriented implementation of CI, such as build design, the integration process, testing and organisational structure. In our work, however, the focus is on ensuring the safety properties of ASSs by analysing hardware-related faults that violate functional safety requirements using CI practice.

The findings in this section show a paucity of investigation into integrating CI practices into the automotive system development process, i.e., system integration test with HIL, and highlight the need for further research in this area. Besides, optimizing the analysis process of the HIL test recordings is a gap in the literature that needs to be addressed. This study proposes a novel approach for applying CI during HIL testing, in accordance with the ISO 26,262 standard and considering the real-time constraints of the SUT on embedded machines.

## Methodology

**Fig. 1:**
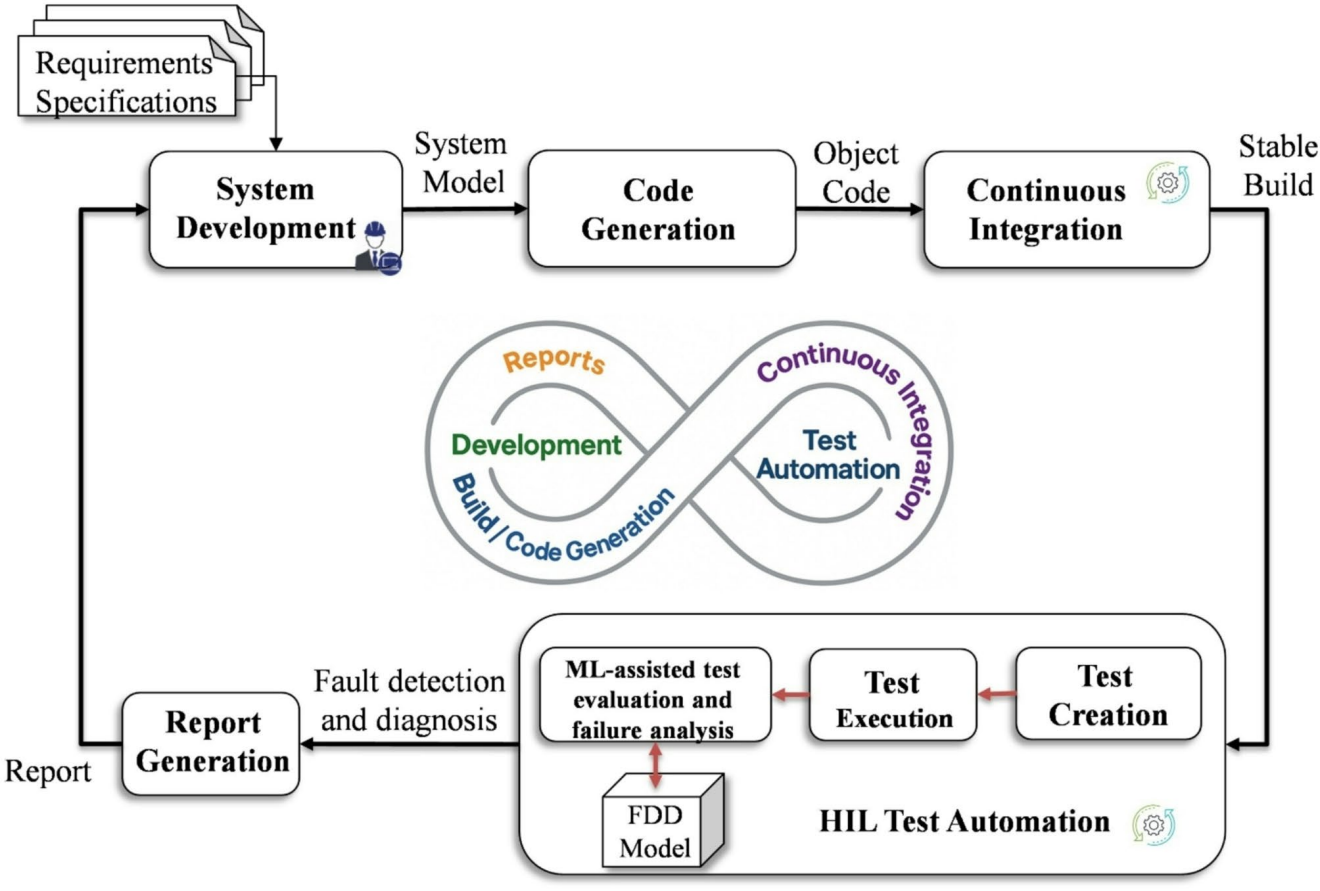
Proposed methodology for ML-assisted test records analysis based on HIL and CI.

This section presents the methodology proposed to address the challenge of failure analysis in test recordings during the validation process of ASSs. Furthermore, the integration of CI practices within HIL tests to enable efficient test automation is also described. The phases of the proposed methodology are illustrated in Fig. 1, including system development, code generation and deployment, CI, HIL test automation and report generation.

### System development

The target system architecture is modelled on the basis of the requirements specifications, which include system objectives, functional and non-functional requirements, performance criteria and all constraints. To this end, Matlab/Simulink^38^, as a modelling environment, is employed to represent the various components of the automotive system, including sensors, ECUs and actuators, and their interactions. Matlab/Simulink is a powerful tool for visual modelling and simulation of complex vehicle systems based on the block diagram environment. This approach facilitates the creation of an executable system model, which accurately represents the data flow and control logic, while also accounting for the closed control loop between the SUT and the plant. Subsequent to the modelling stage, a range of test activities and scenarios are applied to verify the system under various normal and abnormal conditions. This testing approach is referred to as model-in-the-loop (MIL) testing^39^. The employment of MIL testing ensures that the target system architecture fulfills the requirements specification prior to the generation of the source code.

### Code generation

Subsequent to the validation of the system model against requirements and the confirmation of its behaviour as an accurate representation of the intended system behaviour, automation tools such as Embedded Code in Simulink automatically generate object code. This process converts the graphical system model into high-quality source code, e.g., C++, that includes control and data flow and other computational tasks. However, to meet the non-functional properties of the software, e.g., memory consumption or processor load, an optimization process is carried out, including minimizing memory footprint, improving execution speed and implementing inline functions. In this phase, B2B tests are carried out against the behaviour of the original model to ensure the functional correctness of the generated code. Finally, the source code is compiled by the build system into an executable form on the embedded target computer, including the configuration of build options and considering the operating environment.

### Continuous integration

Following the optimization of the executable object code, the integration and build process is initiated automatically in order to ensure the conversion of model changes into executable code. The deployment of CI practices is applied to increase the efficiency of the process by triggering builds in response to changes in version control systems. CI practices include version control management, automated build processes, automated testing, continuous feedback loops and deployment. In this context, Gitlab^40^ and Jenkins^41^ environments are utilized for version control management and build/test process automation, respectively. Thanks to Gitlab’s branching strategies, the code base can be stored in such a way that traceability, change tracking and merging of updates can be achieved seamlessly, and multiple developers can collaborate efficiently. In the event of a change being detected in the repository, the build process is initiated automatically, based on the latest code version. Specifically, the.

compilation of the code, linking and creation of an executable are performed, which allows for immediate feedback on any abnormal issues. Concurrently with the build process, a series of automated tests is run on the new version, with the aim of verifying the functionality and reliability of the code and ensuring that the new updates have not introduced any bugs. To this end, a HIL platform is used, considering the real-time constraints of the embedded target system. As a result of Jenkins running the automated tests, continuous feedback is sent to the developers in the form of a report that includes the build status, test results, and any issues detected. It is evident that this phase plays a pivotal role in mitigating integration issues and fostering collaborative development by providing higher-quality software with a stable build that is verified and ready for system-level HIL testing.

### HIL test automation

In accordance with the ISO 26262-4 clause 7, an experimental test should be conducted during the system integration and test phase. The objective of these tests is to demonstrate that each component of the system interacts correctly and performs only the intended functions. Furthermore, it is necessary to provide a reasonable degree of confidence that the system does not exhibit any unintended functions that could potentially violate a safety objective.

#### Test creation and execution

Once the SUT has been developed, a comprehensive set of testing scenarios is created and executed on the HIL real-time simulation platform. It is noteworthy that the functional requirements and safety goals specifications serve as a fundamental basis for creating the testing scenarios, which cover both normal and abnormal operational conditions. The application software of the SUT is executed on the real ECU, whereas the entire controlled system (plant) is executed on the HIL simulator.

Notably, in certain instances, real physical elements or systems are connected to the HIL simulation in order to consider their effect on the SUT during the validation process.

During the real-time execution, the sensor and command signals between the controlled system and the SUT are transmitted via the Controller Area Network (CAN) bus. In addition, the output of the SUT is monitored and the corresponding system behaviour is logged as a multivariate time series dataset, which is referred to as test records. Subsequently, the generated test records are subjected to analysis by an expert engineer, who determines the causes and nature of the occurring failures based on evaluation criteria. It is noteworthy that in the case of systematic testing with predefined outputs, the test cases (TCs) are evaluated automatically by HIL, which assigns a pass or fail score to the results without interpreting the causes of the failed TCs. Subsequently, the test results, comprising findings, observations, and identified faults, are documented in a report and transmitted to the development team.

#### ML-assisted test evaluation and failure analysis

The proposed approach, grounded in the data-driven DL approach and the FI method, offers an intelligent solution for FDD in test protocols, operating in an efficient manner without the need for human intervention. This, in turn, helps to improve the process of safety and reliability analysis and to support engineers by reducing the time and effort required. The development of this approach was undertaken in two phases. i.e., analysis of system behaviour and generation of representative data sets, development and application of the FDD model.

### System behaviour analysis and representative dataset generation

**Fig. 2:**
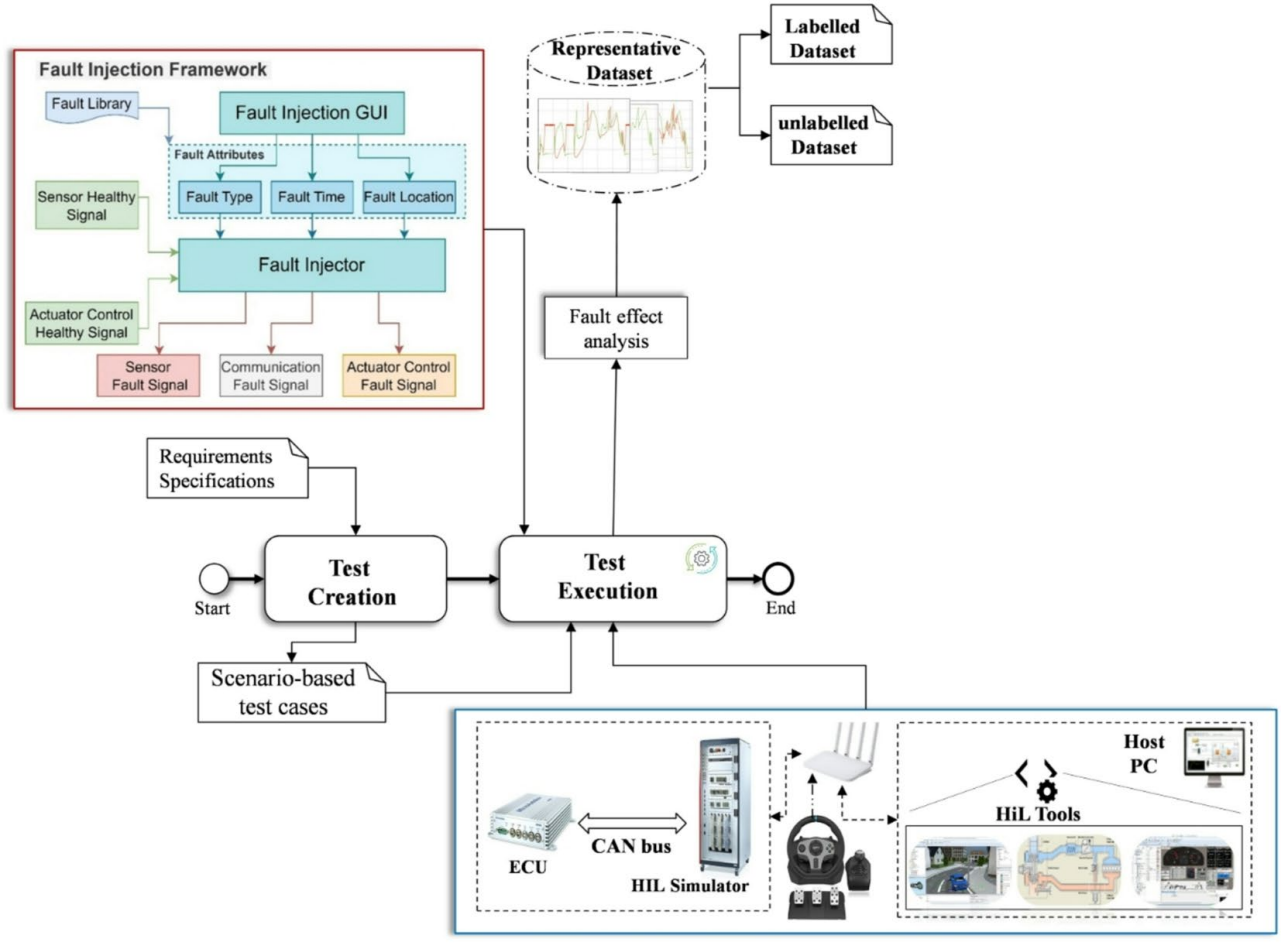
System behaviour analysis and representative dataset generation.

Fault injection (FI) method has been extensively utilized in the automotive industry as a highly effective experimental testing method for the assessment of the system’s resilience, safety, and fault tolerance. The fundamental premise of the concept is to introduce faults artificially into specific components and analyse the system’s response under abnormal conditions. Notably, during the development of ASSs, FI has proven its effectiveness on both sides of the ISO 26,262 V-cycle, as stated in^42^. In this study, a real-time FI framework developed in^43^ was employed to identify critical faults that could lead to violations of safety and functionality. The framework’s novel aspect is its capacity to inject faults in real-time, thereby ensuring the system’s structure as a black box and obviating the need for modeling. In order to perform the FI experiments, it is necessary to identify three different attributes in advance: the fault type, the fault location, and the FI time. The flowchart for analyzing system behaviour in the presence of faults and for creating the data set is shown in Fig. 2. In this study, the majority of sensor-related faults have been considered in the process of system behaviour analysis. These include gain, offset, hard-over, stuck-at, delay, noise, packet loss, drift, and spike faults.

Mathematically, the fault types can be presented as follows:

1 $$Y_f (t) = g_vY_h(t)\: +\:b_v$$

where *Y*_*f*_ (*t*) is a faulty or manipulated signal value, *g*_*v*_ represents the gain value and *Y*_*h*_(*t*) is the healthy or standard signal value *b*_*v*_ represents the offset/bias value.

The list of potential fault locations in the vehicle system is presented in^25^. In this study, the sensors and actuators have been identified as potential points in the system architecture that are susceptible to faults, i.e., locations where faults may occur. The location in the FI framework has been selected based on the assumption that a fault in the component would have a significant impact on the safety and functionality characteristics at the system level. Finally, the time required for the injection of faults into the selected component has been determined based on the critical-safety scenario to be validated. This scenario may include acceleration and deceleration in critical edge cases of the vehicle system. It is noteworthy that the injection process on the aforementioned framework can be achieved either manually or automatically through the use of automation software tools. Once the critical faults have been identified, the corresponding system behaviour under faults is captured as a time series dataset generated in real-time. The data samples collected from the system’s behaviour under critical faults serve as a fundamental representative historical faults dataset for training, validating, and testing the target FDD model. The collected dataset includes both labelled and unlabelled data samples, which will be used to develop the FDD model through supervised and unsupervised learning approaches.

### Machine learning-based FDD model development and deployment

**Fig. 3:**
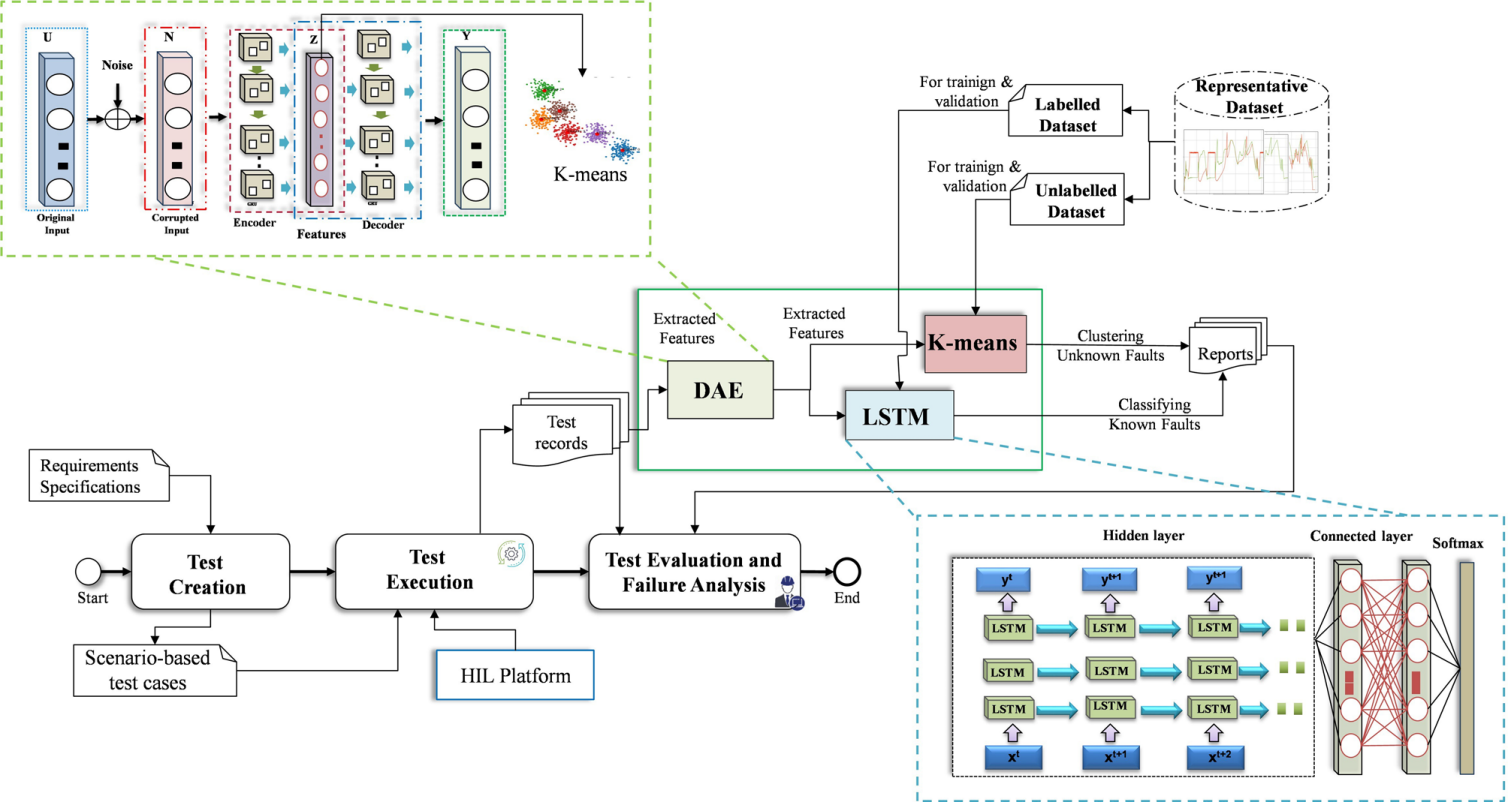
Machine learning-based FDD model development and deployment process.

Due to the fact that the collected dataset from the FI framework is unclean, a preprocessing phase is applied to the data prior to its utilization in the development of target models. The higher the quality of the dataset, the higher the performance of the developed model. This phase serves not only to remove outliers and redundant data but also to correct any missing data samples that may have been introduced during the recording process. However, as a prerequisite, the cleaned data should be converted to a scaled and normalized format, whereby the variables’ values are scaled within the range [0,1]. Finally, the pre-processed data is divided into three portions for training, validation, and testing, with 80%, 10%, and 10%, respectively. In this study, two DL models are developed employing supervised and unsupervised learning techniques to address the issue of FDD of known and unknown faults, respectively, as can be seen in Fig. 3.

In this study, to ensure a high-quality classification and clustering process that is robust against noise, a GRU-based DAE was developed for extracting detailed representative features. Subsequent to the extraction of representative features, the classification and clustering process is initiated.

As a classification problem, a supervised DL approach-based model is constructed to detect and identify the types of known critical single and simultaneous faults. To this end, a multi-label-multi-class LSTM model is proposed based on a historical labeled dataset. The model has been designed to cover both healthy and faulty classes, thereby ensuring an accurate detection and classification process. LSTM is a highly effective DL technique capable of establishing long-term relationships in sequential data, overcoming the limitations of RNN regarding exploding gradient issues. In comparison to other DL structures, such as CNN, MLP, and GRU, LSTM has demonstrated superior performance on large datasets, particularly in the context of supervised classification problems. Therefore, due to its superiority in processing complex nonlinear data in higher dimensions, in this study, LSTM has been selected so that an effective FDD model with high levels of accuracy and reliability can be developed.

The input, the forgetting, and the output gates are three distinct gates within the LSTM cell, which represent the fundamental component of the proposed DL model. In addition, the cell state serves as a memory mechanism that regulates the precise flow of information. This process entails the determination of whether new information is discarded, retained, or reinforced. The mathematical representation of the LSTM cell structure is presented in^44^.

On the other hand, in the case of FDD of unknown faults, unlabelled data is employed to develop the model as a clustering problem using an unsupervised learning approach. However, due to the environmental and operational conditions, noisy data presents a complicating factor in developing a reliable FDD model with an acceptable level of robustness. To address this challenge, DAE has been demonstrated as a promising approach to enhance the resilience of FDD models against noise conditions. The DAE structure comprises two main modules, namely the encoder and decoder, which include input, corrupted, hidden, and output layers. Notably, after the corruption of the original input data with noise, the data is transformed into a low-dimensional representation feature by the encoder module and saved in the latent space layer. This, in turn, contributes to the denoising of the original dataset and the extraction of meaningful features that encapsulate the signal characteristics. It is worth noting that the features extracted in this layer are used in the clustering process on the basis of the k-means technique (Fig. 1). Given that the fault detection process is addressed by the LSTM model, the detection of anomalies in HIL records using DAE has not been investigated in this study. The mathematical representation of the encoder and decoder modules is presented in^45^.

Finally, a multi-level clustering model is developed based on the k-means technique in order to cluster the extracted features from DAE effectively, including the concurrent and single faults. Due to its computational efficiency, scalability, and.

compatibility with the latent features extracted by DAE^46^, the k-means clustering method was selected in our study for the purpose of clustering unknown faults. In particular, the well-structured representation of the fault space provided by DAE is conducive to enhanced clustering performance in the presence of noise, a condition in which the k-means algorithm is particularly effective^47^. Consequently, k-means was selected over alternative clustering methods to circumvent their inherent limitations, which include higher computational costs, sensitivity to noise, and extensive parameter tuning.

The fundamental principle underlying the algorithm is the separation and grouping of features based on their degree of similarity. To this end, the squared distances between the data points and each cluster center are calculated, after which the model is trained so that the calculated distances within the clusters are minimized.

### Report generation

The final process after conducting the HIL tests with the CI pipeline is the generation of test reports, which is done by AutomationDesk^48^ and Jenkins. These reports include documentation of the identified faults, observations, and performance metrics. Subsequent to the completion of the HIL test automation phase, the test reports are uploaded to the Git repository. By doing so, it can be ensured that all team members have access to the test results, which in turn promotes teamwork. Moreover, the decision to store these reports in Git facilitates the monitoring of the software’s evolution, the review of past test data, and its linkage to code changes, thereby providing insights into the performance of the software across iterations. Consequently, the development team undertakes a thorough review of the test reports, identifying areas for improvement and iteratively enhancing the software for continuous improvement.

### Case study and experimental implementation

To validate the effectiveness of the proposed approach, a HIL-based real-time virtual test drive platform is developed and utilized. In particular, a high-fidelity vehicle dynamic system model with traffic provided by dSPACE^49^ is employed as a case study.

**Fig. 4:**
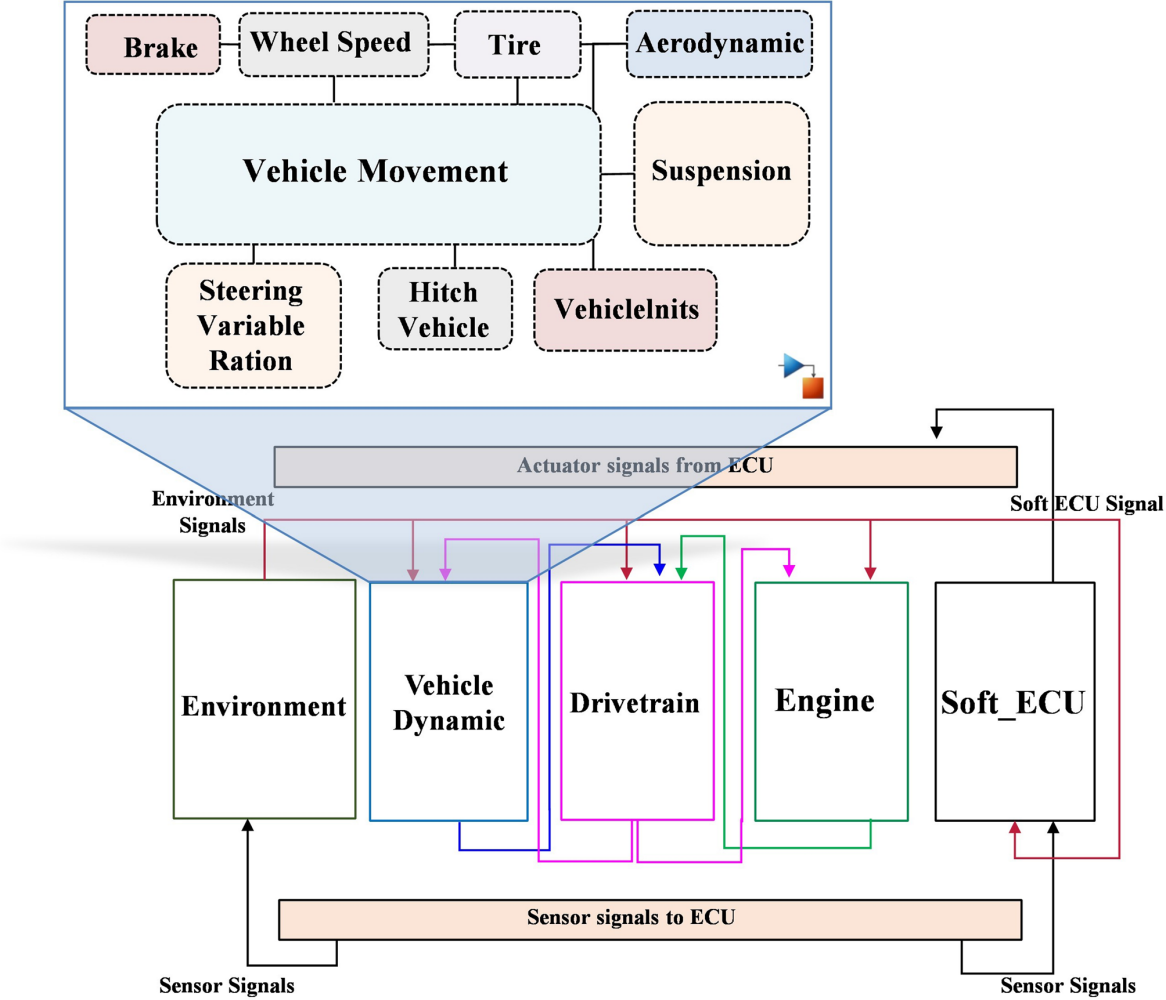
System model architecture of the used case study.

However, to accurately represent the intricate details of the vehicle engine, a sophisticated gasoline engine model has been incorporated into the vehicle model. The case study offers a comprehensive analysis of the vehicle system, including engine, vehicle dynamics, drivetrain, external environment and ECU model, as can be illustrated in Fig. 4. In this study, the ECU model of the gasoline engine has been selected as an SUT and separated to be executed in the target ECU hardware.

**Fig. 5:**
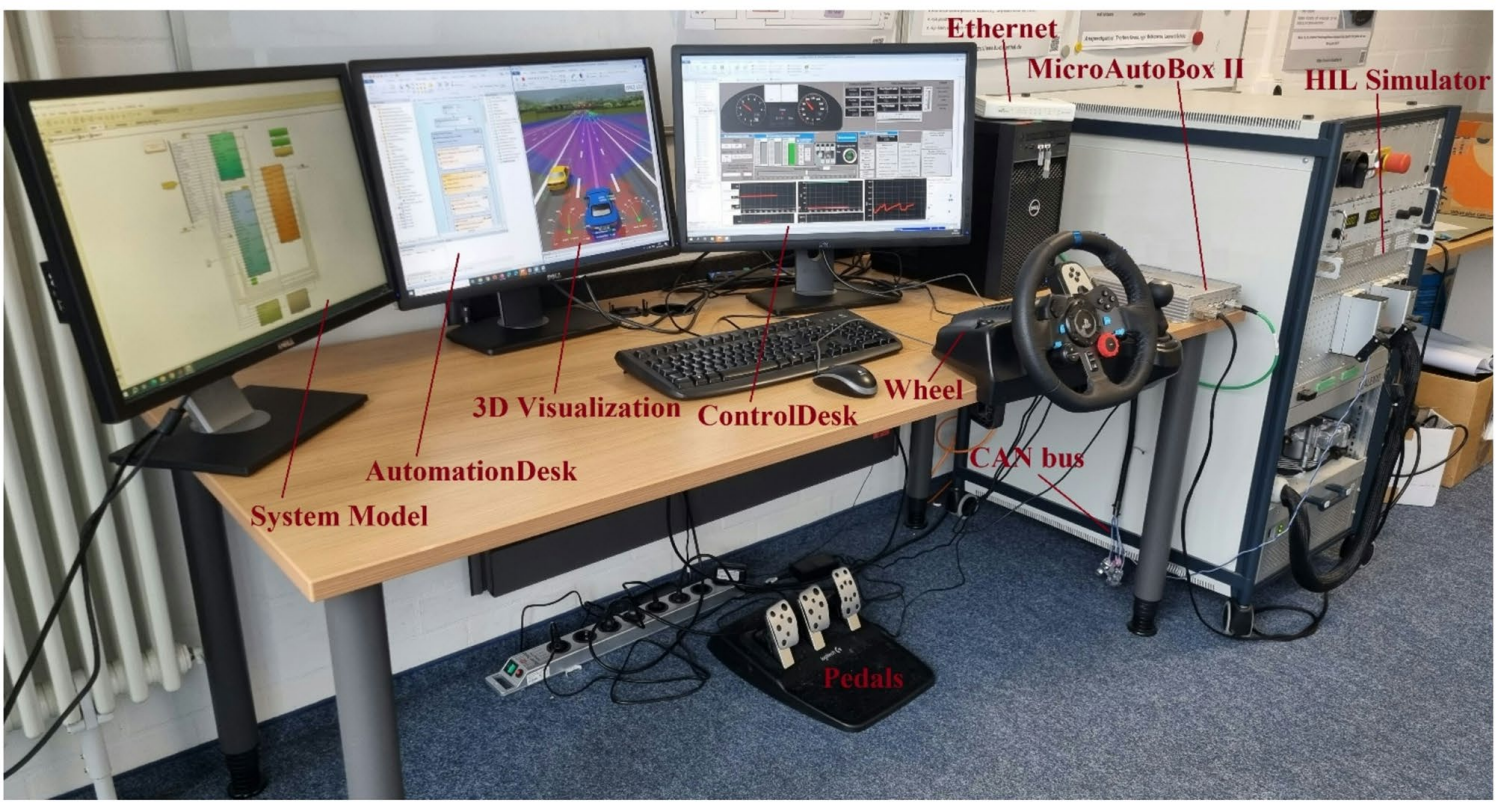
Real-time virtual test drive platform with HIL system.

To establish the connection between the separated ECU and the controlled system (plant), the Real-Time Interface Multimessage Block Set (RTICANMM) has been employed to model the communication interface. The developed RTICANMM model provides access to system variables, which, in turn, facilitate the process of FI programmatically without the need for modelling effort. The generated code from the ECU and plant model is deployed and executed in the real-time system, i.e., in the real ECU and HIL simulator, respectively. In this study, a Rapid Control Prototyping MicroAutoBox II embedded PC (DS1401 base board) with a 900 MHz processor, 6th Gen. Intel^®^ CoreTM i7-6822EQ, 16 MB is employed to emulate the functionality of a real ECU. Whereas the SCAELXIO DS6001 processor board is employed as the real-time processing hardware, in addition to the real steering wheel and pedals, as illustrated in Fig. 5. To ensure the system is capable of operating in a wide range of driving scenarios, a 3D model of the environment has been created, which includes a variety of weather conditions, traffic patterns and road topologies.

### Continuous integration framework during HIL testing

The proposed CI-based validation framework was developed based on Jenkins (LTS 2.414), running on Windows Server (16-core Intel Xeon CPU, 64 GB RAM, Docker-enabled) and set up in a multi-branch pipeline configuration. The Jenkins pipeline is initiated by GitLab commits, thereby automating the build, deployment, and test execution processes. In particular, the management of software builds was undertaken using Gradle. Whilst unit tests are executed via MATLAB tests on a host PC running Windows 10 Pro (64-bit), integration tests via HIL are enabled by means of automatically deploying the validated builds to the target machine. The software MATLAB/Simulink 2021b and dSPACE AutomationDesk (version 6.6-2021B) are utilised for unit and integration tests, respectively.

**Fig. 6:**
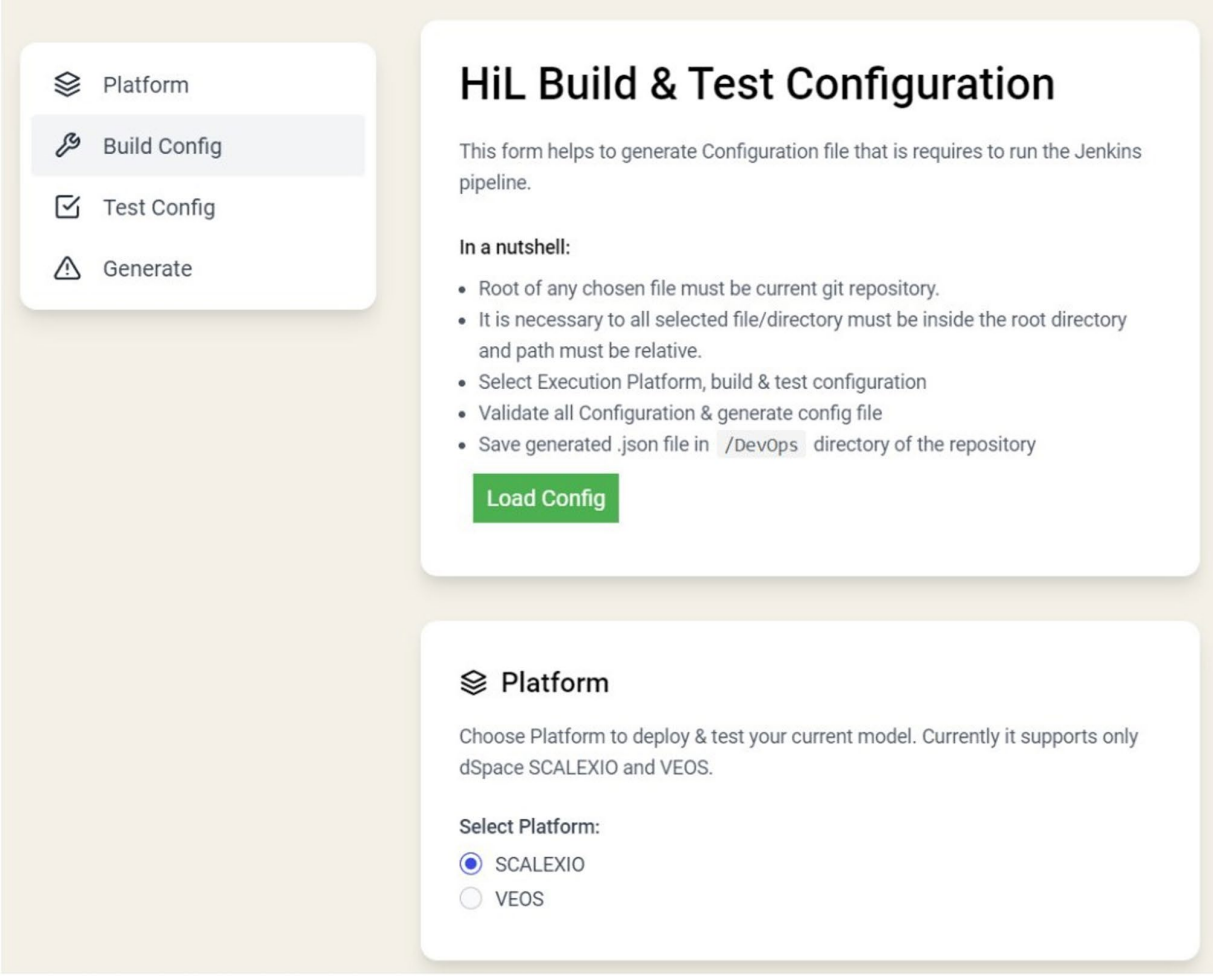
HTML form to generate the configuration of CI framework.

The CI framework presented includes four main stages, i.e., initialization, build, test setup, and outcomes. To simplify this sequence, an HTML form has been crafted to aid in generating the configuration file as can be illustrated in Fig. 6. This form establishes the foundational settings for the build, detailing platform details, locations of model files, Simulink initial commands, paths for VEOS files, AutomationDesk projects, and the extent of reports. Initially, a directory structure is established within the assigned Git repository. This repository is made up of two sections, i.e., the vehicle dynamics model and the AutomationDesk project with the test scenarios. During execution in Jenkins, the produced JSON file is utilized by the Jenkinsfile to guide the pipeline.

In the initial phase, the local codebase is updated by performing a fetch and pull from the GitLab repository, ensuring it reflects the latest commit on the branch. The setup and path configurations needed for building the target platform are sourced.

from BuildTestConfig.JSON, which is created via an HTML form. Proper configuration of the GitLab plugin within Jenkins is crucial, as it streamlines access to GitLab during execution and enables Git operations on the repository. For Jenkins to be triggered remotely, the user’s PC should have a steady internet connection and a fixed DNS for Jenkins hosting. Triggering Jenkins can be done through the standard HTTPS protocol.

The build phase allows the execution of both SIL and HIL tests by running the object code on VEOS or SCALEXIO platforms, respectively. On VEOS, a SIL file is created from the vehicle dynamics model using the MATLAB/Simulink plugins integrated into Jenkins. This SIL file is then uploaded to the target platform. Conversely, ConfigurationDesk is used to generate the build, including setting up the interfaces between SCALEXIO and the target ECU. This phase concludes when the model’s code is generated and deployed onto the target embedded system automatically.

During the test phase, the AutomationDesk tool is employed to run predefined test cases under various conditions. Finally, the pipeline concludes in the results phase, where a ZIP file containing the test reports is stored back in the repository. Additionally, an email notification is sent to the relevant project team members, requesting them to inspect and address any detected issues.

### Dataset description

In addition to the fault-free data samples, the collected dataset includes critical fault data samples resulting from the FI process in real-time. The critical fault samples were determined by analyzing the effect of the injected faults that resulted in a system-level failure in real-time. The accelerator pedal position (APP) sensor and the engine speed (RPM) sensor were selected as locations of the faults due to their significant effect on the vehicle’s safety. Sensor-related fault types, including gain, stuck-at, noise, drift, and delay, were considered in this study as fault types. Finally, according to the standard desired driving scenario, the FI time has been set so that the acceleration and deceleration modes of the vehicle driving can be validated. In particular, the fault types have been injected into the APP and RPM sensors, individually and simultaneously, between 170 and 330 s as transient faults.

**Fig. 7:**
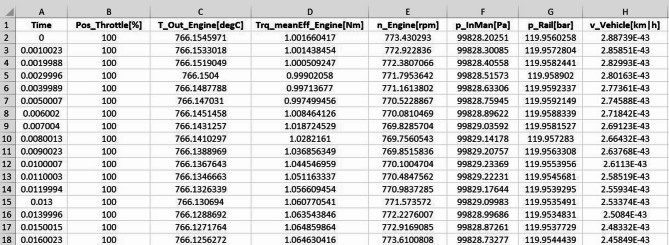
Variables of the Dataset used for FDD model development.

Consequently, the generated dataset contains imbalanced data between healthy and faulty classes, considering the single and concurrent faults. In order to capture the system’s behaviour, a number of variables have been selected and recorded, including throttle position (%), engine temperature (degC), engine torque (Nm), engine speed (rpm), manifold pressure (p.a.), rail pressure (bar), and vehicle velocity (km/h). The selection of system variables was made on the basis of expert knowledge from the fields of automotive engineering and control systems. This was done so that the variables with a high indicative value for engine and vehicle behaviour and sensitivity to sensor-related faults are taken into account. These variables have been recorded with a sampling rate of 0.001 s, as can be observed in Fig. 7. Thus, as a result of the data recording process, besides the healthy class, 15 distinct classes of single and concurrent faulty classes have been collected, including 44,800,000 samples with 2,800,000 samples per class.

### Training and optimization

**Fig. 8:**
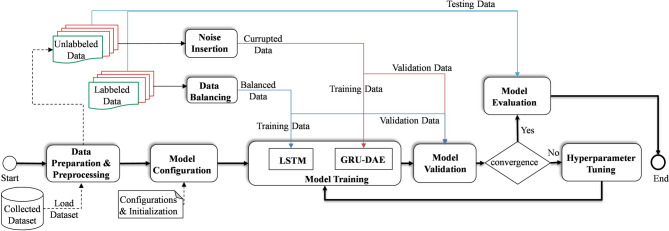
Flowchart of the FDD-based DL models development.

In this study, Google Colab has been employed as an implementation platform for the proposed model, utilizing the TensorFlow framework and Python programming language. The implementation process was conducted in accordance with the four phases of the workflow illustrated in Fig. 8, i.e., data preparation, model configuration, model training and validation, and model testing.

In the data preparation phase, the data undergoes preprocessing, which involves the removal of outliers and redundant information, scaling, and normalization. Furthermore, the dataset is divided into three distinct subsets, with 80% allocated for training, 10% for validation, and 10% for testing. It is noteworthy that a balancing technique, namely random undersampling, is applied to the labelled data in order to achieve an equal ratio between the fault-free and fault classes. Besides, a Label Power Set (LPS) was employed to perform the labelling process, with the objective of assigning fault classes to the corresponding data. Conversely, the unlabeled data is corrupted by the addition of a specific level of Gaussian noise for the purpose of training the DAE model. In this study, Gaussian noise was added to the data at four levels: 3%, 6%, 8%, and 10%. In the second phase, the parameters of the architecture are identified, including the hyperparameters of the model, such as the batch size, the number of layers, the optimizer, the epoch, the learning rate, and the activation function. Once the model parameters have been set, the training process commences. During this phase, the model is trained in accordance with the defined epoch. Upon completion of each training iteration, the validation process is initiated by evaluating the model’s performance based on the calculation of the loss function and the utilization of validation data. Consequently, based on the calculated loss function resulting from each training, the model is optimized by updating the hyperparameters until convergence is achieved. In this study, a grid search mechanism has been employed in the training and validation process in order to identify the optimal hyperparameters in an efficient manner. The optimal hyperparameters of the LSTM and DAE models, which provide the best performance, can be illustrated in Table 1.

## Results and discussion

The performance of the proposed LSTM model has been evaluated using an unseen testing dataset based on three quantitative evaluation measures, i.e., precision, recall, and F1-score. In contrast, the mean-square-error (MSE) and Davies–Bouldin (DBI) scores have been employed for the assessment of the DAE and K-means models, respectively.

### GRU-DAE performance for denoising and feature extraction

The performance evaluation of the DAE shows high achievement in terms of low reconstruction error between the input and output. In particular, the MSE of the proposed model with noise-free data is 0.0073. This result demonstrates the applicability of the model in reconstructing the input data and extracting detailed features from time series data. To illustrate the superiority of the proposed model, the evaluation results have been compared to those of other DAE structure, i.e., ANN-based AE^50^ and CLSTM-based AE^51^. The proposed model exhibits high performance under varying levels of noise, with an average MSE Of 0.0444. Nevertheless, as the noise level increased, the model’s performance declined. Conversely, the CLSTM-based DAE exhibits suboptimal performance with elevated MSE values across varying noise levels. Furthermore, the substantial computational burden associated with the CLSTM-DAE’s complex architecture represents a significant challenge for real-time applications. It is also noteworthy that the size Of the training data has an impact on the performance Of the developed model. Table 2 illustrates that the inclusion Of a limited number Of classes in the faulty data leads to a high performance Of the DAE with an average MSE Of 0.043. As the number Of fault classes included in the dataset increases, the model’s performance declines. This phenomenon can be attributed to the complexity and similarity between the extracted features from the fault data samples. Nevertheless, even under the most extreme conditions Of noise and the inclusion Of all fault types, the performance Of the proposed model remains within an acceptable range, with an average MSE Of 0.044.

**Table 1:** Optimal hyperparameters Of GRU-based DAE and LSTM architecture.

Hyperparameter	GRU-based DAE	LSTM
GRU Layers	4	-
LSTM Layers	-	3
Dense Layers	2	-
GRU Unit	384	-
Latent Space	7	-
Hidden Layer Size	-	512
Batch Normalization Layer	2	-
Epochs	850	60
Batch Size	128	1024
Learning Rate	0.0001	0.001
Activation Function	tanh	tanh
Optimizer	adam	adam

**Table 2:** MSE of DAE under various levels of noise and data size.

Noise Level	25% of data	50% of data	75% of data	100% of data
Noise free	0.0099	0.0152	0.0242	0.0073
Noise 3%	0.0232	0.0238	0.0264	0.0234
Noise 6%	0.0399	0.037	0.0496	0.0421
Noise 8%	0.0492	0.0462	0.0541	0.0504
Noise 10%	0.0598	0.0582	0.066	0.0618

### LSTM performance for fault detection and classification

In the proposed approach, representative features are extracted from DAE and then classified using LSTM layers. This, in turn, contributes not only to superior performance compared to conventional methods but also to robustness against noise.

**Fig. 9:**
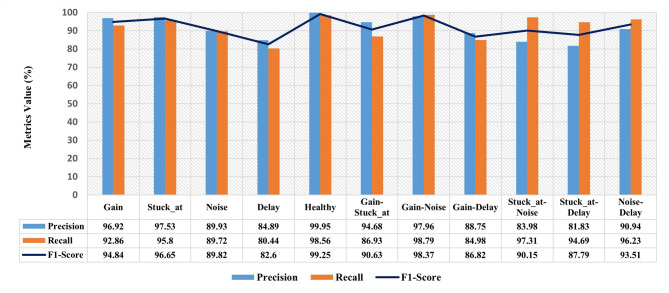
Proposed model performance for single and concurrent faults classification.

From Fig. 9, it can be demonstrated that the model is capable of accurately detecting and classifying single faults as well as the fault-free class, with an average F1-score of 92.63%. Nevertheless, the model’s capability to classify the features of delay fault is limited, with an F1-score of 82.6%. The reason behind this result is the low sensitivity, which results in a high rate of falsely classified features being identified as negative instances. Notably, although the model exhibits a low recall score in classifying simultaneous faults, it shows an acceptable performance in identifying the simultaneous fault types, with an average F1-score of the classes of 91.21%. Due to the distinguishable fault features, the highest accuracy has been achieved in the identification of Gain-Noise faults and Noise-Delay faults, with an accuracy of 98.37% and 93.51%, respectively. To demonstrate the effect of integrating DAE in the proposed approach on the diagnosis process, the classification accuracy for single and concurrent faults has been compared with that from the traditional individual method. The performance of an integrated DAE feature extraction with LSTM-based classification model and standalone classifiers, i.e., models not incorporating a DAE, for the classification of known faults. The comparison was executed under controlled experimental conditions, employing the same data sets to illustrate the relative advantage of the integrated model.

As demonstrated in Table 3, it is evident that the proposed approach has led to a substantial enhancement in the classification performance, particularly in the presence of both single and concurrent faults. This improvement is characterised by an F1-score of 92.63% and 92.36%, respectively, highlighting a notable enhancement in the accuracy of classification.

**Fig. 10:**
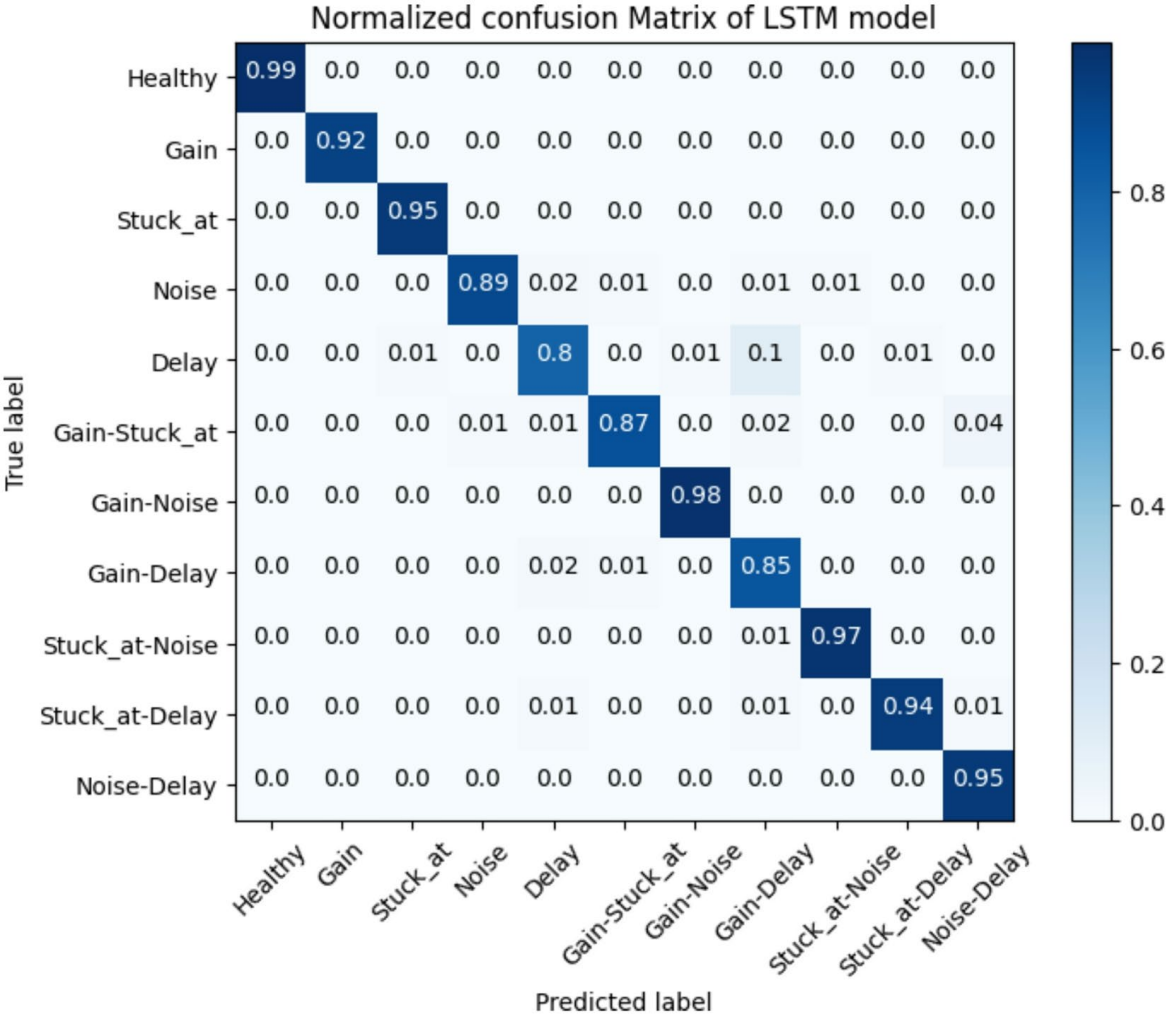
Normalized confusion matrix of the proposed LSTM model.

In order to provide a comprehensive demonstration of the classification performance of LSTM, a normalized confusion matrix including single and concurrent fault classes has been presented. As illustrated in Fig. 10, some samples of Delay fault ´have been incorrectly identified as Gain-Delay due to the similarity of single and concurrent fault patterns. Notwithstanding, the model shows a high performance in classifying the Gain-Noise and Stuck_at-Noise. Consequently, the proposed model exhibits remarkable average classification accuracy for both single and concurrent fault types, outperforming other traditional methods. To substantiate the superiority of the proposed model, the classification results have been compared to those of related works. Table 4 demonstrates that the model outperforms other state-of-the-art models with an average accuracy of 91.85%.

**Table 3:** Comparison analysis of proposed model performance with different stand-alone classifiers in terms of F1-Score (%).

Model	Single Fault	Simultaneous Faults
SVM	81.17%	77.93%
DT	79.48%	73.84%
MLP	90.66%	88.5%
1D-CNN	92.7%	87.44%
Stand-alone LSTM	89.32%	90.74%
Proposed Model	92.63%	92.36%

**Table 4:** Comparison between the results of the proposed method and other related works.

Reference	Method	Accuracy
^31^	PCA-multiclass SVM	84.93%
^32^	PCM	81.49%
^24^	DNN + 1D-CNNs	89%
^52^	MLBCL-EE	87.57%
^53^	HML-KNN	85.07%
^54^	SSAE	87.61%
Proposed work	DAE-LSTM	91.85%

### K-means performance for fault detection and clustering

The extracted features from DAE are then clustered and separated into several clusters using k-means algorithms according to the similarity via four-level clustering. The level 1 clustering differentiates between fault-related and healthy features. At level 2, the faulty features are organized into clusters based on their corresponding components. At levels 3-a and 3-b, the faults that occur in specific components are grouped according to their type. Finally, at level 4, the features of the concurrent faults are clustered.

**Fig. 11:**
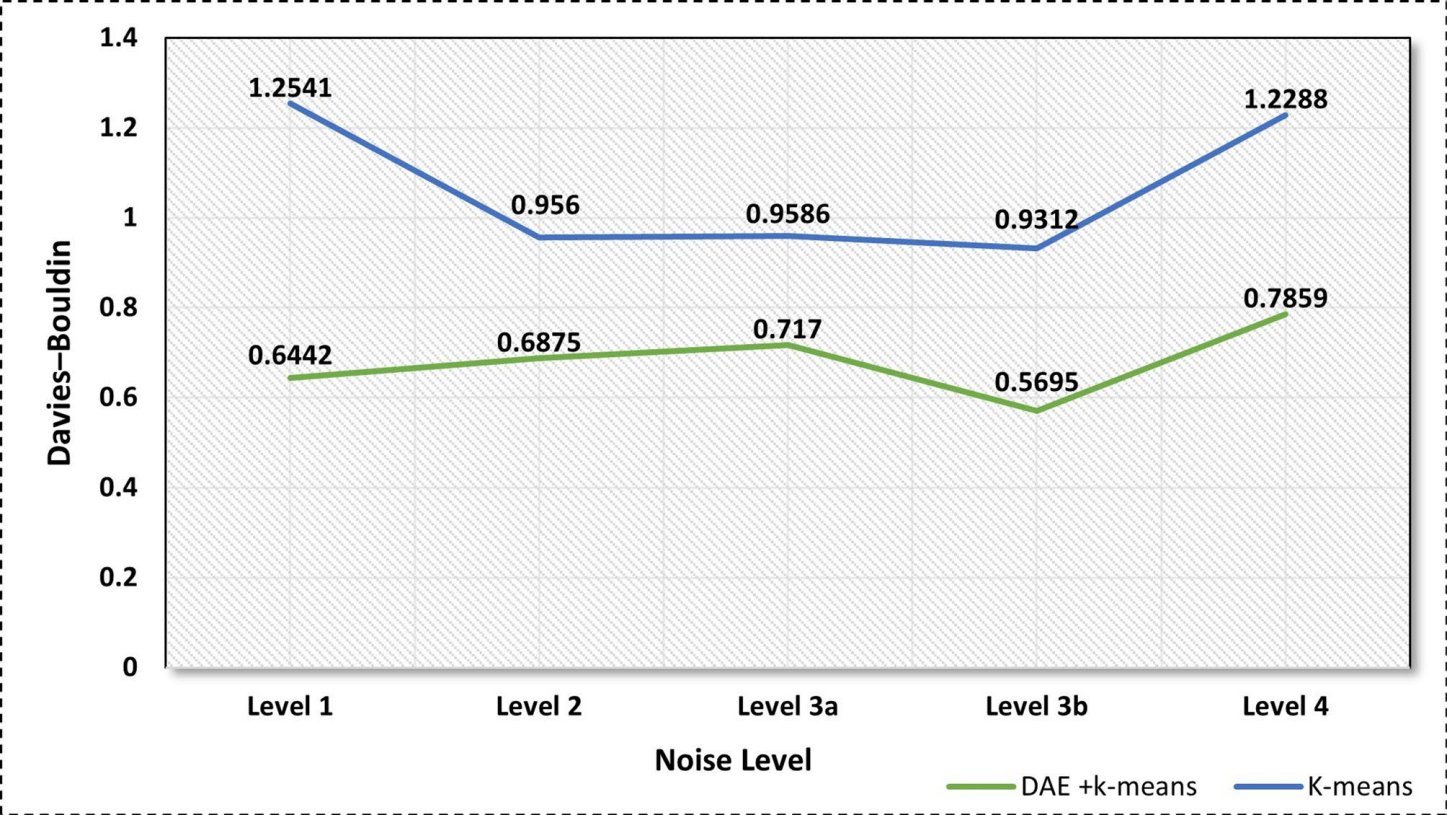
Proposed model performance for single and concurrent faults clustering.

In order to demonstrate the effect of using DAE on the clustering performance, the validation results of the proposed model have been compared to those of a traditional k-means model. Figure 11 illustrates that the clustering model’s performance has been enhanced by a 0.3849 value in terms of DBI. It can be reasonably mentioned that the low value of DBI indicates a superior performance of clustering tasks. Notably, the proposed model demonstrates remarkable performance with a DBI score of 0.7859, which is significantly lower than the DBI value of 1.22 achieved by traditional clustering. The quantitative evaluation metrics demonstrate that the proposed model has achieved high performance in the detection task of single faults in unlabeled data. The precision, recall, and F1-score are 99.17%, 95.23%, and 97.16%, respectively. Furthermore, the concurrent faults detection accuracy of the model with an F1-score of 95.38% indicates the reliability and robustness of the proposed model under noisy conditions. It is notable that the clustering performance exhibits high accuracy in clustering faulty features in level 3b with and without DAE, with a DBI score of 0.5695 and 0.9312, respectively. This can be attributed to the fact that the fault-related features of the system components at this level, i.e., the APP, are more discernible and exhibit less complexity in comparison to the faulty features of other components.

#### Development process performance with CI practices

**Fig. 12:**
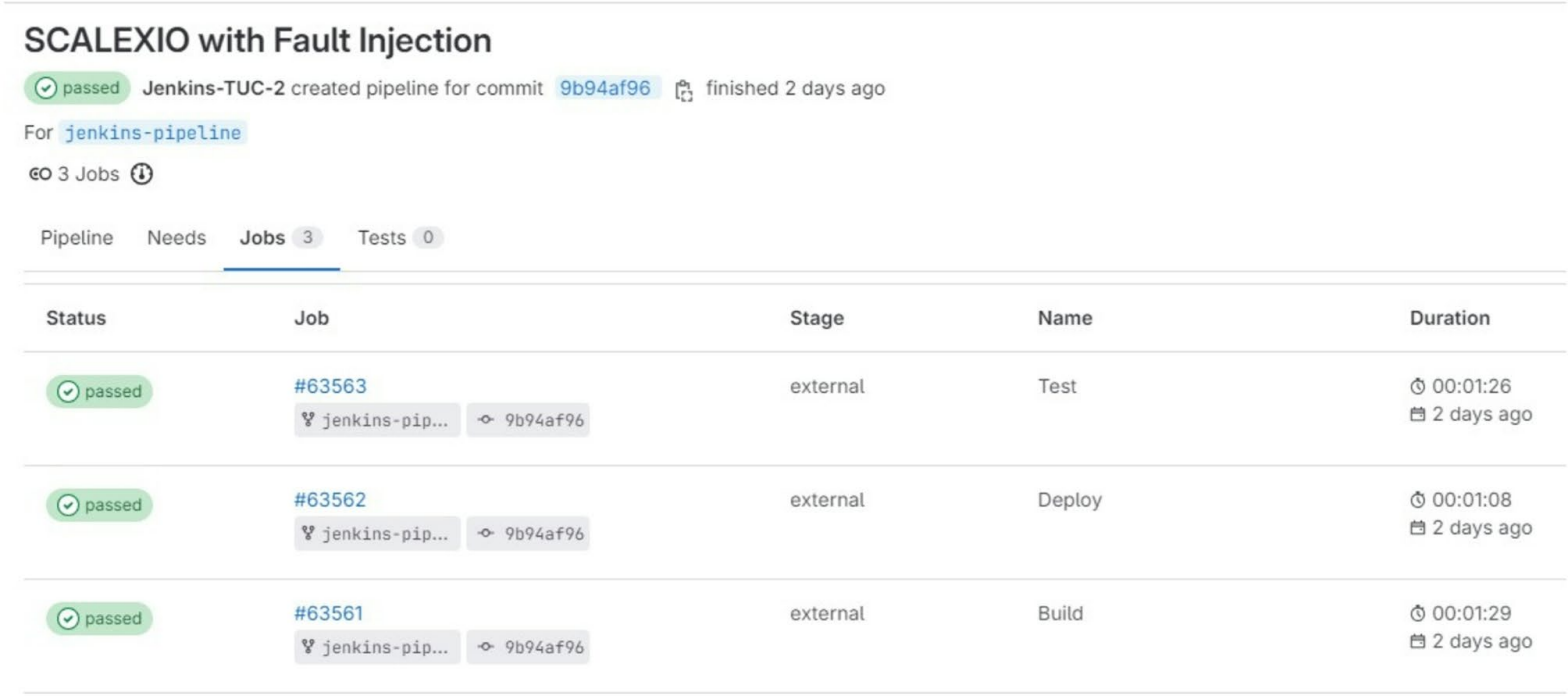
Gitlab pipeline stages.

The GitLab plugin enhances the ability to manage stages within Jenkins by offering details such as the stage name, execution time, and duration, as depicted in Fig. 12. The Name column displays the specific stage as identified in the Jenkins file. This functionality provides developers with the ability to monitor the execution status directly from GitLab, thus enhancing accessibility and clarity in pipeline monitoring without needing to access Jenkins itself.

Conversely, Jenkins features a stage visualization capability, where stages are represented as consecutive blocks for each execution instance, allowing custom labels instead of the standard job ID. Figure 12 illustrates how the GitLab pipeline stages, which offer detailed information on all previous executions, including timestamps and the number of new commits associated with the current execution. The stages, defined in the Jenkins file, begin with the checkout process and conclude with the post-action phase. Each block displays the total duration of its respective phase, facilitating quick evaluation of the time demands of various stages.

**Fig. 13:**
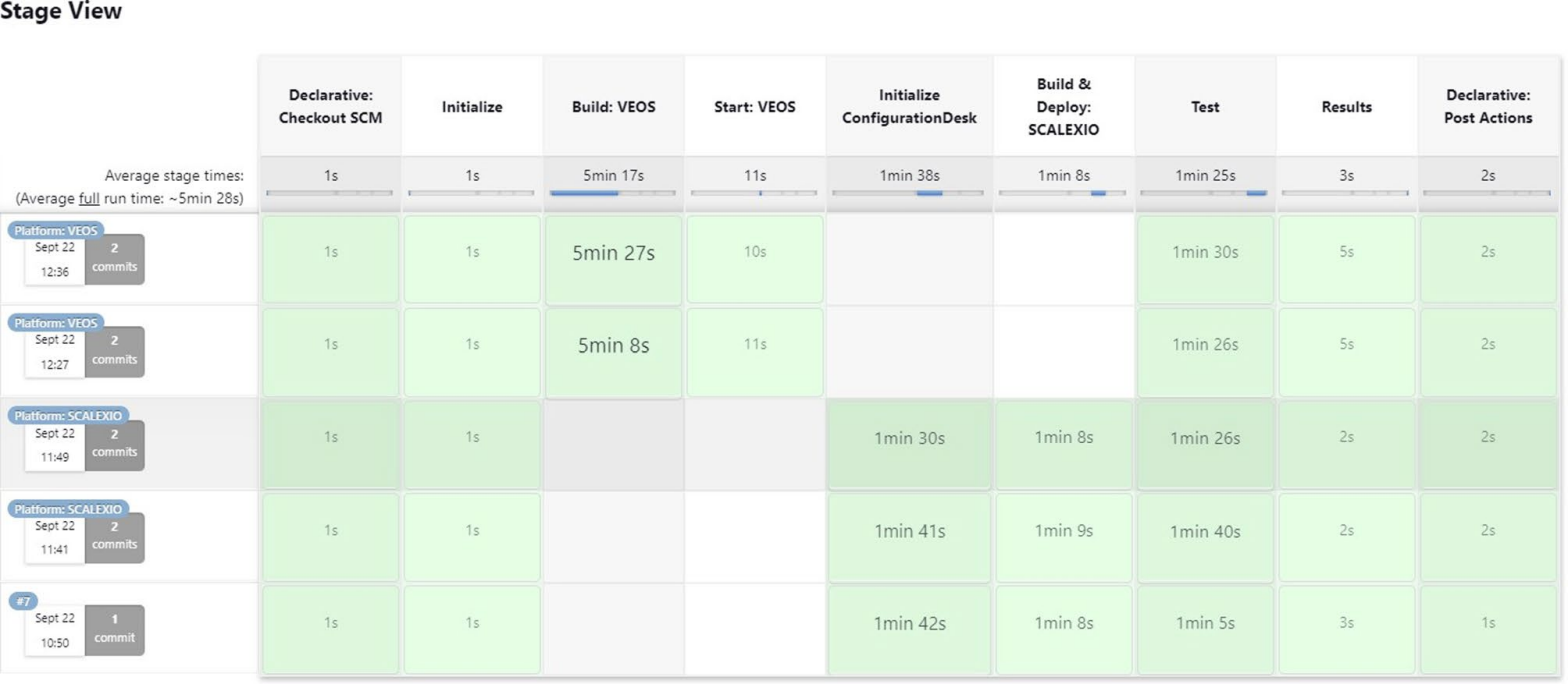
The results of applying CI practices with different testing platforms.

The metric used for evaluation captures the duration from the initiation of a development task to its deployment in the production environment. This measure offers insights into the performance of both the development and deployment workflows. Analyzing the timing throughout various phases of this study is critical for assessing how effectively the proposed CI approach manages the HIL testing process. By evaluating the durations across different systems and phases, the impact of automation and enhanced workflows on speeding up development activities can be identified. The timing measurement begins when a commit is pushed to Git and ends when Jenkins logs the completion of the entire process. In contrast to the time-consuming manual steps that are characteristic of conventional workflows and require step-by-step meticulous attention, the proposed methodology significantly reduces manual intervention and accelerates the overall process, minimizing human effort and time. A comparison of the time required by different software integration methods is shown in Fig. 13.

The evaluation process includes two rounds, with each conducted on a distinct target platform, i.e., SCALEXIO and VEOS. The process takes 8 min on SCALEXIO, whereas on VEOS, it requires 9 min. It is worth noting that the additional duration for VEOS is attributed to the extra steps needed by the VEOS player application, not by any shortcomings in efficiency.

Traditional methods often exceed 15 min and involve more complex execution protocols, whereas our proposed approach demonstrates a notable reduction in processing time. It is important to acknowledge that both the test cases in this study are simpler than those typically used in real-world production environments, underscoring the potential time reductions achievable through DevOps practices. Table 5 provides a comparison between the conventional and new approaches. Although the time gap is not significant in this setup, it could be substantially larger in an actual production environment.

### Conclusion and future work

This article presents a novel CI-enabled validation approach for automotive embedded real-time systems, providing continuous software development through iterative cycles. The CI practices have been integrated into HIL test activities to perform change tracking and traceability in an efficient manner during the validation of at the system integration and testing phase. At the core of the CI framework, i.e., test automation phase, ML-based intelligent failure analysis is proposed to automatically detect and identify the cause of a failure based on a representative faults dataset and data-driven DL methods. To this end, real-time FI has been employed to analyse the system’s behaviour and identify the critical faults that result in the violation of safety goals. The data collected was used to develop a supervised learning-based LSTM model architecture for the purpose of detecting and classifying the known single and concurrent faults. Moreover, the k-means algorithm has been developed to detect and cluster unknown single and concurrent faults. The GRU-DAE is used for denoising and extracting meaningful features, while the LSTM and k-means methods are used for classifying and clustering the extracted features from the DAE within the latent space, respectively. The proposed model has demonstrated considerable efficacy in identifying faults within an unseen dataset derived from a real-time virtual test drive, exhibiting an average accuracy of an F1-score of 91.85% and a superior performance compared to current FDD methods. Moreover, the integration of DAE with k-means contributes to enhancing the clustering performance of unknown faults in the presence of noise, with a low MSE and DBI, i.e., an average of 0.044 and 0.68, respectively. Moreover, the evaluation results of the gated recurrent unit (GRU)-based DAE demonstrate a notable superiority in terms of low MSE compared to other structures, such as the ANN and CLSTM-based DAE. Moreover, the findings indicate that the implementation of the proposed CI framework can lead to a substantial reduction in manual intervention and a significant acceleration of the overall process, thereby minimising human effort and time. The achieved accuracy of the proposed model and the efficiency of CI practices contribute not only to the improvement of safety and reliability of the ASSs, but also to the reduction of cost, time, and effort during the development process of complex dynamic systems.

**Table 5:** Comparison of the required time for various software integration approach.

Software Integration Approach	Required Time	Execution
CI with HIL SCALEXIO	8 min	automated pipeline
CI with HIL VEOS	9 min	automated pipeline
Manuel integration	15 min	manual step-by-step procedure

A further enhancement to the proposed failure analysis module is to ensure the interpretability and explainability of the decision-making process. In this context, the integration of formal causality-based methods, such as structural causal models (SCMs), directed acyclic graphs (DAGs), and counterfactual reasoning, represents a promising extension.

## Supplementary information

Below is the link to the electronic supplementary material.

Supplementary Material 1

## Data Availability

Data available on request due to restrictions.
